# Transplanted neurons integrate into adult retinas and respond to light

**DOI:** 10.1038/ncomms10472

**Published:** 2016-02-04

**Authors:** Praseeda Venugopalan, Yan Wang, Tu Nguyen, Abigail Huang, Kenneth J. Muller, Jeffrey L. Goldberg

**Affiliations:** 1 Neuroscience Program, University of Miami, Miami, Florida 33136, USA; 2 Shiley Eye Center, University of California, San Diego, California 92093, USA; 3 Department of Physiology and Biophysics, University of Miami Miller School of Medicine, Miami, Florida 33136, USA; 4 Byers Eye Institute, Department of Ophthalmology, Stanford University, Stanford, California 94303, USA

## Abstract

Retinal ganglion cells (RGCs) degenerate in diseases like glaucoma and are not replaced in adult mammals. Here we investigate whether transplanted RGCs can integrate into the mature retina. We have transplanted GFP-labelled RGCs into uninjured rat retinas *in vivo* by intravitreal injection. Transplanted RGCs acquire the general morphology of endogenous RGCs, with axons orienting towards the optic nerve head of the host retina and dendrites growing into the inner plexiform layer. Preliminary data show in some cases GFP^+^ axons extending within the host optic nerves and optic tract, reaching usual synaptic targets in the brain, including the lateral geniculate nucleus and superior colliculus. Electrophysiological recordings from transplanted RGCs demonstrate the cells' electrical excitability and light responses similar to host ON, ON–OFF and OFF RGCs, although less rapid and with greater adaptation. These data present a promising approach to develop cell replacement strategies in diseased retinas with degenerating RGCs.

## Abstract (web summary)

Replacing lost retinal ganglion cells is a potential treatment for optic neuropathies such as glaucoma. Here, the authors show that transplanted donor RGCs can successfully integrate into a mature host retina *in vivo*, projecting axons along the intact visual pathway and responding to light stimuli.

## Graphical abstract

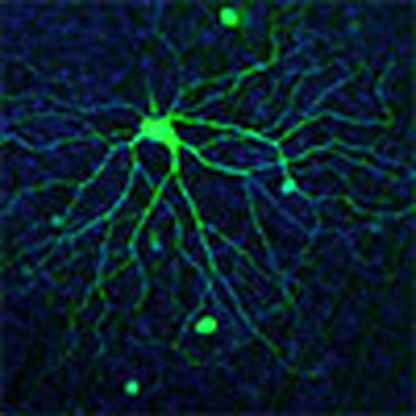

Because central nervous system (CNS) neurons generally are not replaced over the mammalian lifespan, transplantation of replacement neurons to restore neural circuits is increasingly attractive for recovery from CNS damage or disease. Many diseases of the retina, a part of the CNS, impair vision through death of retinal cells. For instance, glaucoma and other optic neuropathies lead to death of retinal ganglion cells (RGCs), causing permanent loss of vision. The retina presents an accessible, structured CNS environment to study functional integration following transplantation. There has been success in transplanting cells into the outer retina to replace degenerating photoreceptors as therapy for diseases like retinitis pigmentosa or age-related macular degeneration^1^,^2^,^3^,^4^, but cell connectivity and physiology of photoreceptors or retinal pigment epithelium are different than those of RGCs, which are CNS projection neurons with complex patterning. Transplanted cells may be neuroprotective for RGCs after optic nerve insult^5^; for example, transplantation of stem cell-derived retinal progenitors and embryonic retinal progenitors can improve optic nerve regeneration^6^ and visual function^7^ in RGC-depleted animals. Less progress has been made in cell replacement strategies for RGCs lost in advanced disease states. There is little evidence that transplanted retinal precursors or differentiated RGCs can integrate into the host retinal circuitry and thereby contribute to improved retinal function. The present experiments were therefore designed to assess the potential of cell replacement within the retina. We show that transplanted primary RGCs survive, migrate and make functional synaptic connections after *in vivo* transplantation into adult, uninjured host eyes.

## Results

### Survival and growth of transplanted RGCs in the host retina

**Figure 1:**
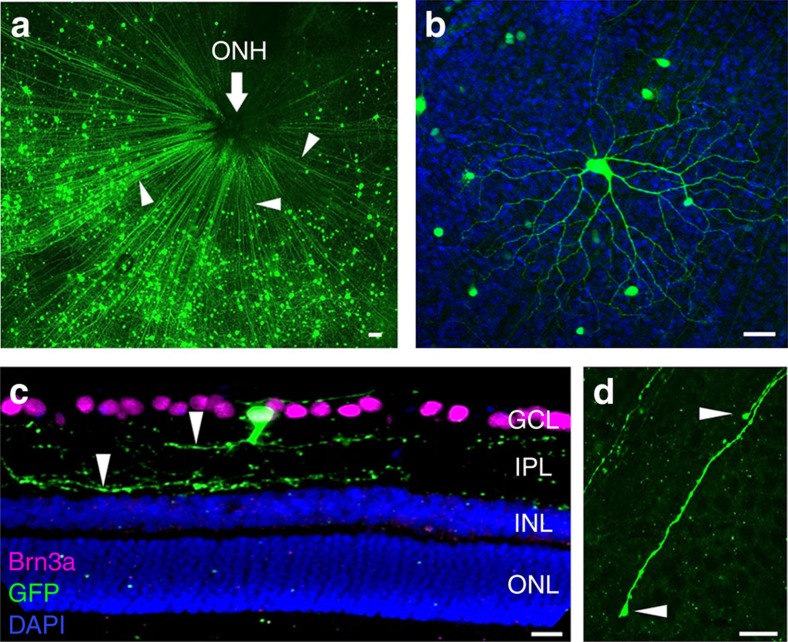
Morphology of transplanted GFP^+^ RGCs in the host retina. (**a**) After 3 weeks, transplanted GFP^+^ RGCs were observed in host retinal explants with putative axons (arrowheads) extending towards the optic nerve head, ONH. (**b**) Elaborate dendrite architecture of donor RGCs with morphologies often seen in known subtypes of endogenous RGCs. (**c**) In retinal sections from host eyes, GFP^+^ RGCs were seen within the host ganglion cell layer, GCL, extending neurites (arrowheads) into the inner plexiform layer, IPL. (**d**) One week after transplantation, some GFP^+^ axons in the host retinal explants were observed with growth cone-like terminal endings (arrowheads) in the nerve fibre layer. Scale bars, 25 μm.

We previously studied the short-term transplantation of primary RGCs after intravitreal injection^8^, but longer-term assessment of axon and dendrite growth and electrophysiological integration past 7 days post-transplantation had not been attempted. To address this, 99.5% pure green fluorescent protein positive (GFP^+^) RGCs^9^,^10^ were transplanted bilaterally or unilaterally from early postnatal mice into the vitreous space of adult Sprague–Dawley rats, ages 1–3 months. In some transplantations (*n*=15 of total 152), transplanted (donor) RGCs migrated through the nerve fibre layer into the host ganglion cell layer (Fig. 1a). There was variability in the number of GFP^+^ RGC donor cells visible in the host retinas, ranging from 50 to more than 2,000 GFP^+^ RGCs per explanted host retina. Host eyes were fixed and treated with fluorescently labelled anti-GFP antibodies and, when biocytin was injected intracellularly, with fluorescent streptavidin to see more clearly the morphology of the transplanted cells. Axon-like projections extended from the individual GFP^+^ RGC cell bodies into the nerve fibre layer and towards the host optic nerve head (ONH) among unlabelled, host retinal axons. Many GFP^+^ RGCs had elaborate dendritic trees (Fig. 1b and Supplementary Fig. 1). Most (70%) transplanted RGCs were found to be positive for Brn3a, similar to the known distribution of Brn3a in rodent RGCs (Supplementary Fig. 2). In retinal cross-sections, dendrite-like neurites extended into the host inner plexiform layer (IPL; Fig. 1c). Thus, the transplanted RGCs had acquired three-dimensional morphologies similar to host RGCs and, in laminating within the IPL, appeared to be capable of integrating morphologically with the host retina. At a shorter, 1 week time point, growth cone-like structures directed towards the ONH were observed at the ends of transplanted RGCs' GFP-labelled axons (Fig. 1d and Supplementary Fig. 3), indicating new, directed growth from transplanted cells.

In 70 of 152 experiments, parallel cultures of donor cells were plated concurrently with transplantations on poly-D-lysine- and laminin-coated tissue culture plates in serum-free RGC growth medium^9^. Of these 70 experiments, successful transplantation *in vivo* occurred in 6 of the 14 eyes for which parallel cultures of donor cells survived *in vitro*, whereas transplantation failed in all 56 eyes for which parallel cultures of donor cells did poorly (*P*<0.0001 by Fisher's Exact test, two-tailed; Table 1). The overall success rate with parallel cultures, 6 of 70, was comparable to the 9 of 82 with which parallel cultures were not made. Thus, although there may be several variables contributing to successful transplants, it is important to use donor cells that survive well.

**Figure 2:**
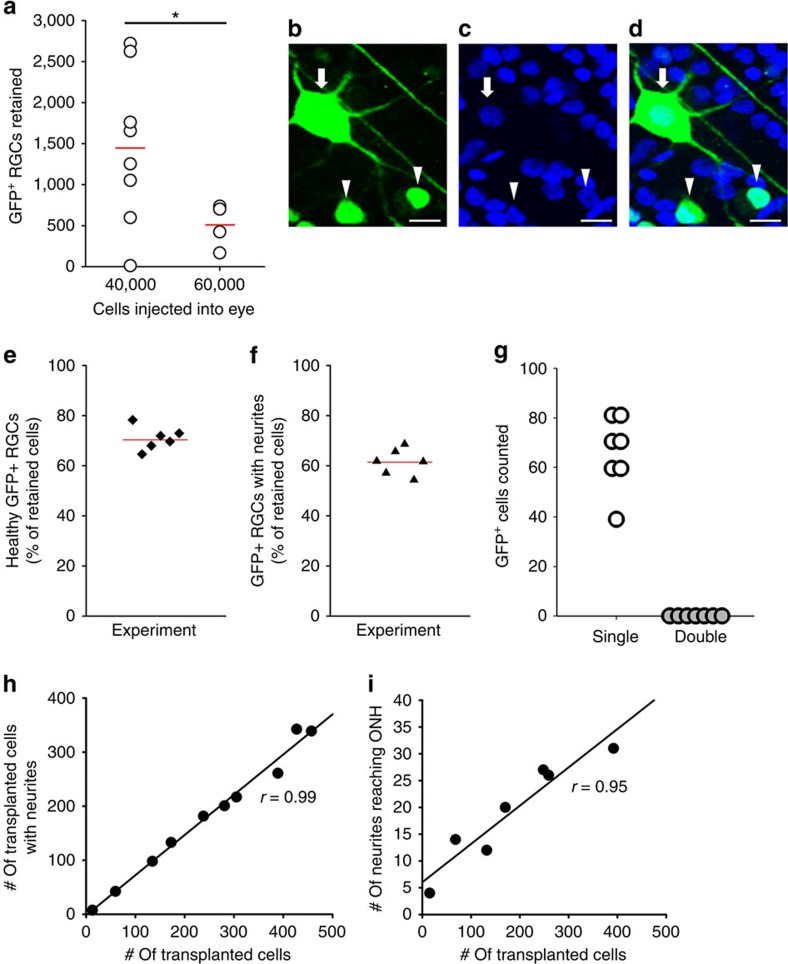
Transplanted GFP^+^ RGCs survive and extend neurites in the host retina. (**a**) Retention of donor GFP^+^ RGCs 3–4 weeks after successful intravitreal transplantations, with significantly different mean values shown as red lines (*P<0.05 by *t*-test, two-sided). (**b**–**d**) Representative images from recipient, DAPI-stained retinal whole mounts showing nucleus morphologies (blue) and the corresponding green GFP-merged images of donor RGCs; examples of punctate, unhealthy nuclei (arrowheads) and large, circular, healthy nuclei (arrow) are marked. (**e**) Based on DAPI morphology, 70% of the retained GFP^+^ RGCs were judged to be healthy, and (**f**) over 60% extended axon-like neurites in the host retina. (**g**) Of nearly 500 GFP^+^ RGCs counted, all had single nuclei and none with double or fused nuclei were observed in the host retinas (*n*=7). (**h**,**i**) For GFP^+^ cells, the fraction of cells with neurites was constant (*r*=0.99), independent of the number of cells transplanted; the number of neurites reaching the optic nerve head (ONH) within the same quadrant increased linearly with cell number (correlation coefficient *r*=0.95), indicating that neurite growth too was not influenced by transplanted cell density. Scale bars, 20 μm.

Variable numbers of GFP^+^ RGCs were visible in the recipient retinas removed from the host eyes; for the two quantities of cells injected, there were paradoxically more cells on average when fewer RGCs were injected into the eye (Fig. 2a). The highest retention of donor cells was ∼7%, but values closer to 1% were more typical (Fig. 2a). Owing to the low number of successful transplants at each time point, it was not possible to determine whether the number of retained donor cells declined with time after transplantation, but of the cells retained after 1–4 weeks, more than 70% of GFP^+^ RGCs in the retina were healthy, as judged by 4,6-diamidino-2-phenylindole (DAPI)-stained nucleus morphology (Fig. 2b–e). More than 60% of GFP^+^ RGCs observed in the host retinas extended axon-like neurites after 1–4 weeks (Fig. 2f). Donor RGCs also extended dendrite-like processes and the number of transplanted cells with visible dendrites was significantly higher than those without visible processes; almost 80% of the retained GFP^+^ RGCs had dendrite-like processes, some elaborate. Although there is little precedent for it, we addressed whether fusion of transplanted cells with host cells could explain the RGC-like morphologies and functional properties acquired by GFP^+^ transplanted RGCs. In whole-mounted retinas that allowed clear observation of individual nuclei with DAPI staining (Fig. 2b–d; *n*=7 retinas), we counted nuclei associated with individual GFP^+^ cells to evaluate the presence of fused cells. Similar images were obtained from sectioned retinas (Fig. 1c). Using confocal microscopy of whole mounts, we found only single nuclei and no double nuclei (Fig. 2g), indicating that none of the labelled cells resulted from cell fusion.

The number of GFP^+^ cells with visible neurites was linearly proportional to the density of GFP^+^ cells in a given quadrant (Fig. 2h), as was the number of GFP^+^ neurites that reached the ONH (Fig. 2i), suggesting that donor cells grew neurites and axon-like projections independently of donor cell density. The regression line correlating density of retained donor cells and extension of neurites up to the ONH (Fig. 2i) could be affected by bundling of axons at higher densities, lowering the numbers of neurites counted; indeed, the correlation coefficient was greater (*r*=0.96) when the highest density was omitted.

### Morphology and dendrite stratification of transplanted RGCs

**Figure 3:**
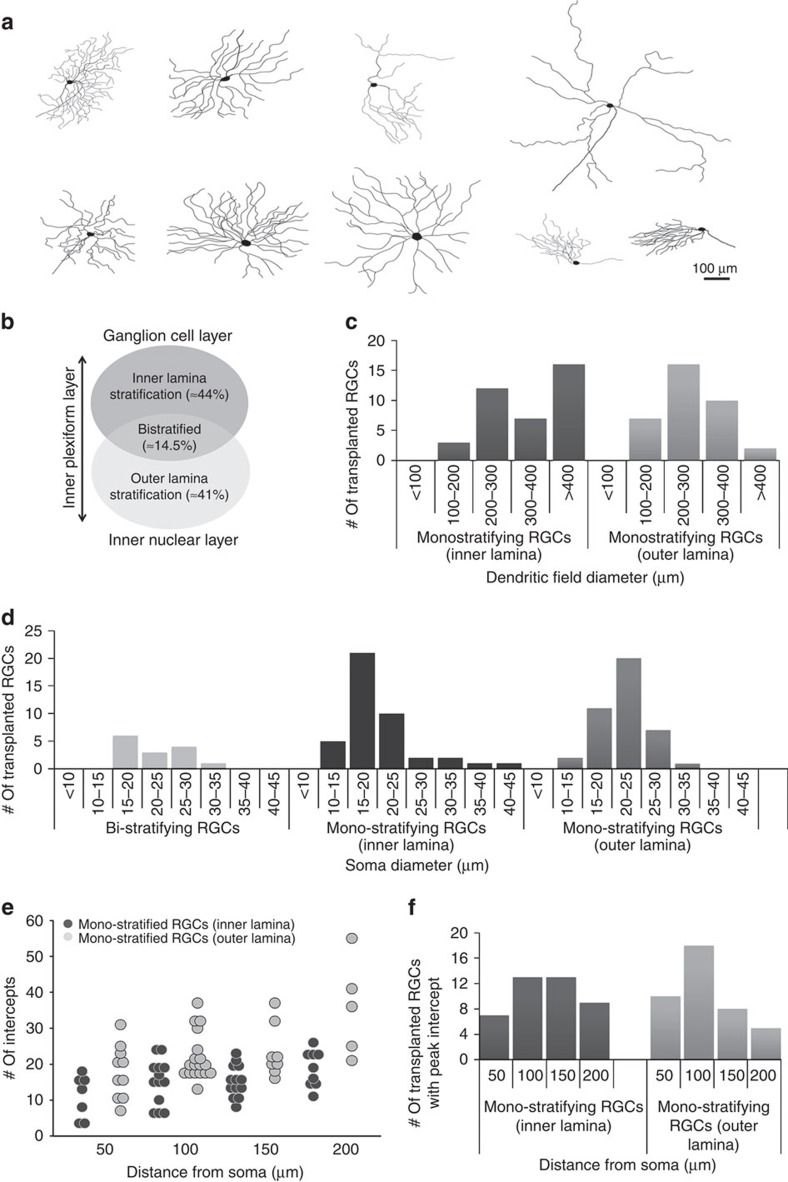
Transplanted RGCs exhibit varied morphologies and dendrite architecture. (**a**) Representative tracings of GFP^+^ transplanted RGCs demonstrating the types of morphologies seen 3–4 weeks post transplantation. (**b**) Schematic representation of the distribution of mono-stratifying and bi-stratifying transplanted RGCs observed in the host retinas. (**c**–**f**) Comparison of distribution of different morphological properties in transplanted RGCs: dendritic field diameter (**c**), soma diameter (**d**), peak intercepts in each cell against distance from cell bodies by Sholl analysis (**e**,**f**).

Transplant recipient retinas were stained with anti-GFP antibodies to show donor RGCs including the architecture and stratification of their dendrites. RGCs with brightly labelled dendrites and distinct arbours were imaged using confocal microscopy, traced using Adobe Photoshop and their morphologies analysed. Similar to previous studies of labelled RGCs and RGC subtypes^11^,^12^, dendrite arbours had varied shapes and extent (Fig. 3a). Similar numbers of transplanted donor RGCs stratified in the inner or outer laminae of the IPL (over 40% each), and fewer (almost 15%) were bistratified (Fig. 3b). Monostratifying-inner, monostratifying-outer and bistratifying RGCs were separately analysed, measuring their dendritic field diameter (Fig. 3c), soma diameter (Fig. 3d), distance of peak intercepts by Sholl analysis from the cell body (Fig. 3e,f). We found that RGCs stratifying in the inner lamina tended to have large dendritic fields (>400 μm) compared with RGCs stratifying in the outer lamina, where most dendritic field diameters ranged within 200–300 μm. In contrast, the majority of RGCs stratifying in the inner lamina had smaller cell bodies (15–20 μm) compared with most of the RGCs stratifying in the outer lamina (20–25 μm); bistratified RGCs showed a broader distribution across these sizes (Fig. 3d). As one measure of complexity, we counted the number of intercepts made by dendrite branches at different distances from the cell body using Sholl analysis. Most of the cells had peak intercepts between 100 and 150 μm from the cell body in both inner and outer lamina-stratifying RGCs. These data highlight the large variability in dendrite architecture of transplanted RGCs, suggesting that either various morphological subtypes survive the transplantation process, or that transplanted cells take on a variety of subtype morphologies when integrating into the local host environment.

After transplantation, donor RGCs extended individual axons radially along the nerve fibre layer towards the ONH (Fig. 1a). To test whether these axons traversed the optic nerve and extended towards the various brain targets of RGCs, we sectioned and stained the optic nerves (*n*=3) and brains (*n*=1) from a small subset of transplanted animals. Preliminary results show that at 4 weeks after unilateral transplantation, numerous GFP^+^ axons were visible in the optic nerve sections, some extending into the optic chiasm (Supplementary Fig. 4a). We did not observe any GFP^+^ axons in optic nerves from uninjected eyes. Using volumetric analysis (*n*=6), we estimated that on average, ∼140 axons grew into the host optic nerves at 1 month post transplantation. In all the sections examined (*n*=6, 2 sections for each of the 3 optic nerves), we did not notice obvious branching or growth cones indicating termination within the optic nerve. Axons were also seen crossing to the contralateral optic tract at the optic chiasm, growing up the optic tract and terminating prominently in the dorsal and ventral lateral geniculate nucleus and the superior colliculus (SC; Supplementary Fig. 4b,d). Comparison with AAV-GFP-labelled RGC axon tracts and terminals in the brain (Supplementary Fig. 4c,e) showed that axon terminals from transplanted donor cells exhibit exuberant growth and extend into deeper layers of the SC, behaviour that is not characteristic of the mature visual system. Donor RGC axon terminals were observed in the anterior portion of the host SC closer to the midline, the expected area of projection based on the ventral temporal localization of the transplanted RGCs in the corresponding host retina^13^. In all the brain sections examined, no GFP^+^ axons were visible in brain regions outside the visual pathways. These preliminary data indicate that some of the donor RGCs that survive and extend axons are capable of reaching usual RGC targets in the brain, but may overshoot their local addresses, at least at 4 weeks.

### Synapse formation and light responses in transplanted RGCs

**Figure 4:**
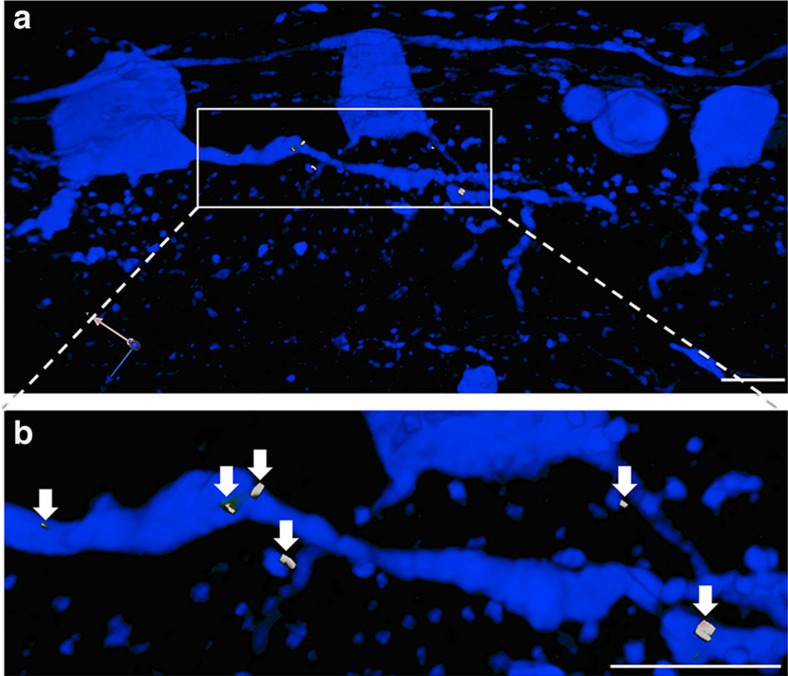
Transplanted RGCs form multiple synapses within the host retina. (**a**) Section from recipient host retina 4 weeks post transplantation showing GFP^+^ transplanted RGCs in blue and synaptic puncta in white. (**b**) Magnified view of a portion of a GFP^+^ dendrite with visible synaptic puncta (white, arrows). Scale bar, 25 μm.

**Figure 5:**
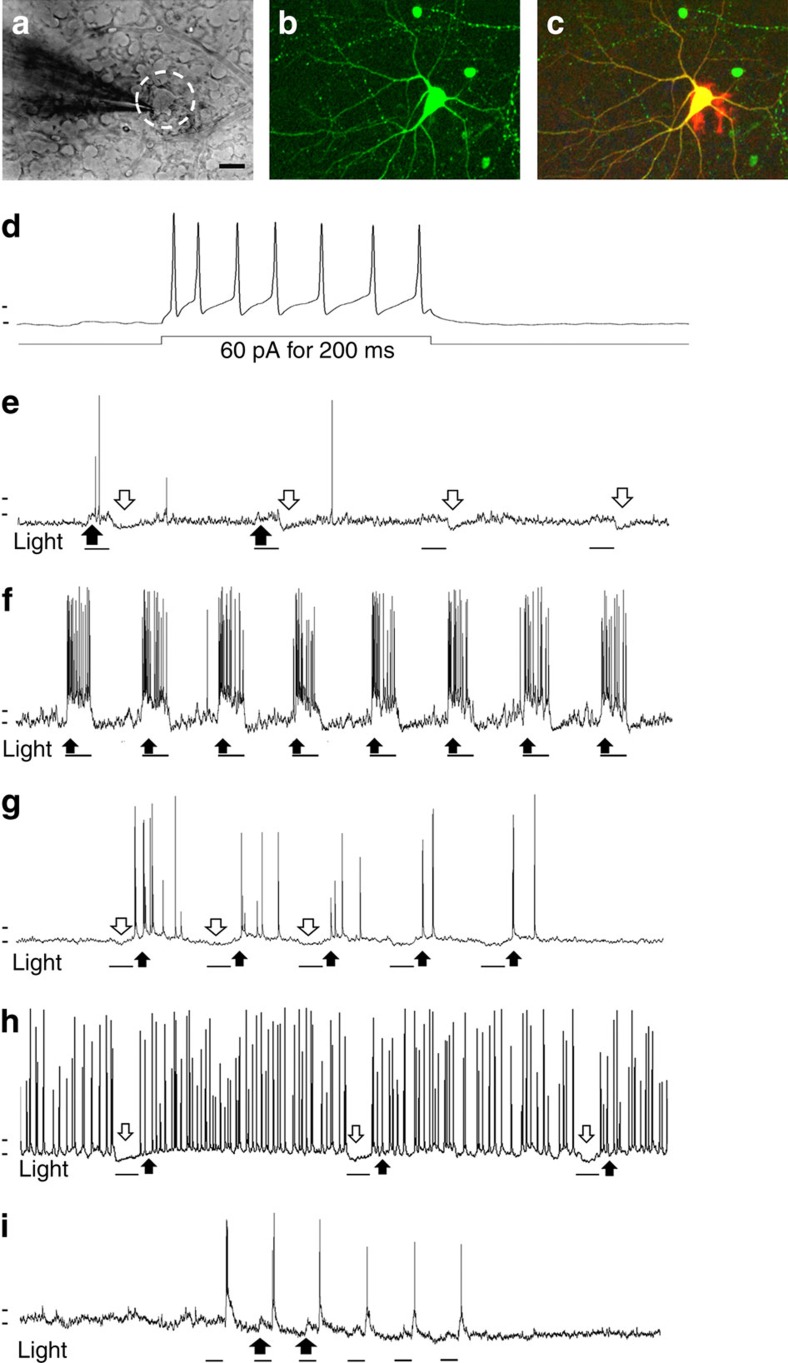
Transplanted GFP^+^ RGCs respond to light stimulation of the host retina. (**a**–**c**) Retina during whole-cell patch clamping of a transplanted cell within the host retina, viewed under infrared illumination with differential interference contrast optics (IR-DIC) (**a**), and after fixation with the recorded cell labelled with GFP in green (**b**) and with biocytin in red (**c**) Scale bar, 25 μm. (**d**) Transplanted GFP^+^ RGCs exhibit electrical excitability when stimulated with injected current (60 pA for 200 ms). (**e**,**g**,**i**) Light-evoked responses recorded from different GFP^+^ RGCs in host retinas after 2–4 weeks include an ON response (**e**), an OFF response (**g**), and an ON–OFF response (**i**). (**f**,**h**) Example traces of light-evoked responses from endogenous, host ON RGC (**f**) and OFF RGC (**h**) exhibiting robust firing with similar stimulation. Light flashes are 1 s; horizontal marks before the traces positioned at −50 and −60 mV. In all traces, black arrows indicate depolarization/action potential firing and white arrows indicate hyperpolarization, black bars under each electrical trace correspond to duration of light stimulation.

Sections of recipient retinas with transplanted cells were stained for GFP and the synaptic markers synaptophysin and PSD95. Synaptic puncta within the sections were identified as regions of co-localization between these pre- and post-synaptic markers using Volocity software (Perkin-Elmer). Synaptic puncta were identified associated with GFP^+^ dendrites from transplanted RGCs visible within the 30-μm sections that were analysed (Fig. 4a,b and Table 2), suggesting that the transplanted RGCs made morphological synapses with the host retina following intravitreal injection. To test for functional synapses upon the transplanted cells, electrical recordings were made from them at various times after transplantation. For recording, host retinas were acutely explanted and biocytin-filled patch pipettes applied to donor cells, identifiable by their GFP fluorescence (Fig. 5a,b). The recorded patched cells, filled with biocytin, were fixed and stained using labelled avidin to show their extent and labelled anti-GFP^+^ antibody to confirm their identity as donor RGCs (Fig. 5c). Observed at 1 week, donor cells were electrically excitable, firing action potentials in response to depolarizing current stimulation (Fig. 5d). However, we found that the minimum current needed to elicit action potentials was ∼80 pA for transplanted RGCs compared with ∼20 pA for host RGCs (*n*=5 each; *P*<0.05 by Student's *t*-test, two-sided). Spontaneous action potential firing and synaptic events were observed in these cells, indicating communication with other cells after transplantation.

Brief flashes of light were used to test whether the transplanted RGCs responded to stimulation of the host retinal explant and had become functionally connect to it. Acutely explanted host retinas were dark-adapted and viewed under infrared illumination during whole-cell patch clamping of GFP^+^ donor RGCs. ON-, OFF- and ON–OFF-type responses were observed in different donor RGCs (Fig. 5e–g, respectively; *n*=5 GFP^+^ RGCs, from three separate host retinas), similar to the known diversity of physiological responses for RGCs^14^,^15^,^16^. ON-like responses were recorded from two donor cells in separate retinas at 1 and 2 weeks post transplantation, and in each case the response adapted to the first stimulus, demonstrating high susceptibility to adaptation of the ON response (Fig. 5e). This was in sharp contrast to robust light responses observed in host RGCs under similar stimulation conditions, with little adaptation between stimuli (Fig. 5e). Another of the recorded GFP^+^ RGCs exhibited OFF-type response, hyperpolarizing at light onset and firing multiple action potentials at light offset, with some progressive weakening with repetitive stimulation (Fig. 5f). This was different from the action potentials in host OFF RGCs at light offset, which were maintained, without appreciable adaptation to repeated stimuli (Fig. 5f). ON–OFF-type light responses were elicited from two other GFP^+^ RGCs 2 weeks after transplantation, again showing adaptation for the ON response, yet responding to each light offset (Fig. 5g). Neither the short response latencies nor the ON or OFF responses matched expected properties for intrinsically photosensitive RGCs^17^,^18^,^19^,^20^. In addition, OFF-type responses elicited from some transplanted cells make it less likely that the recorded responses came from melanopsin-containing RGCs. These results indicate that transplanted primary RGCs retain electrical excitability, make synapses and connect functionally with the host retina. However, these synapses may be weaker than the established synapses of host RGCs, as indicated by their adaptation and less robust firing.

## Discussion

We found that after transplantation *in vivo,* purified primary RGCs survived within the host eye, migrated through the nerve fibre layer and the inner limiting membrane, and established themselves within the ganglion cell layer. There they grew dendrite- and axon-like neurites, forming three-dimensional structures resembling normal adult RGCs. The cells also made synapses with the host retina as evidenced by synaptic marker staining and their electrophysiological activity, both spontaneous and in response to light stimulation of the explanted host retina. Thus, the synapses upon donor RGCs from the host retina are functional, allowing them to receive visual input from the presynaptic circuitry.

There were several distinctions between the transplanted cells and RGCs resident in the retina. First, the weak and labile light responses in transplanted RGCs compared with endogenous RGCs were like developing, immature synapses, with significant adaptation^21^. Second, in the host retina explanted within 7 days of transplantation, growth cone-like structures were visible at the ends of GFP^+^ axons that extended towards the ONH of the host retina, indicating new growth. In addition, in preliminary experiments, we found GFP^+^ axon growth in the host optic tract as well as axon terminals in the lateral geniculate nucleus and SC of the host animals (Supplementary Fig. 4). These axon termini were not like mature host RGC termini and had more exuberant targeting reminiscent of early development and, in the SC, even overshot the retino-recipient layers.

These data argue against a hypothesis that transplanted cells fused with the host. Cell fusion has been reported for bone marrow-derived blood cells and oligodendrocytes^22^,^23^,^24^ but only once for neurons, and under conditions quite different than in the present case^25^. Attempts at distinguishing donor mouse RGCs from host rat neurons using various antibodies against species-specific Thy1 antigens were inconclusive owing to cross-reaction between the closely related species. Potential experiments to conclusively rule out the possibility of fusion could include labelling donor RGCs with EdU (5-ethynyl-2′-deoxyuridine) through injections into the pregnant mother before transplantation. We also found that different morphological subtypes appeared to survive transplantation. The smaller fraction of bistratified donor RGCs resembled the distribution seen in developing mouse retinas rather than that in adults^26^. This further strengthens the case against cell fusion as an explanation for our results. Thus, transplanted RGCs shared features of morphology, patterning and physiology of developing RGCs.

The few cell transplant strategies targeting the inner retina have used stem cells or progenitor cells for neuroprotection in disease models of RGC death^5^,^6^,^7^. Although functional recovery may result from neuroprotection, stem cell-derived purified RGCs may also be donors for cell replacement therapy. An important future direction for this model will be to test whether a diseased environment will be as receptive to transplanted primary neurons as an undamaged retina, in addition to comparing different donor cell types. Our data from an *ex vivo* transplant model showed that *in vitro*-derived RGCs received fewer morphologic synapses than primary RGCs^8^. Engineered scaffolds might also aid targeting of donor cells to the retina and promotion of synaptic connectivity. Biodegradable, electrospun scaffolds can target axon growth from RGCs^27^,^28^, and retinal sheets enhance transplantation in the subretinal space^29^,^30^.

Cross-species transplantation strategies are often studied in retinal cell therapies, and a lack of inflammation or immune rejection is usually attributed to the immune-privileged status of the retina^31^,^32^. In our experiments, mouse RGC donor cells survived in the recipient rat retina and did not elicit frank inflammation, similar to findings using human induced pluripotent stem cells and mesenchymal stem cells^3^,^5^,^33^. Whether combining retinal cell transplant with anti-inflammatory drug application improves survival or integration remains to be tested. Although our measured efficiencies were low, the quality of cell integration, at least in these uninjured retinas, suggests that a significant degree of efficacy is possible. Future experiments could be aimed at addressing the survival and function of specific labelled RGC subtypes.

Although more detailed studies are needed to assess the frequency and time course of this occurrence, our preliminary experiments provide tantalizing evidence for donor RGC axons traversing the host optic nerves and terminating in appropriate brain targets. Axon guidance and targeting in the developing retina and visual pathway have been studied extensively, and it is generally believed that axon guidance is tightly regulated developmentally^13^,^34^,^35^. However, some guidance cues, such as ephrin expression in the SC, may be preserved past developmental stages^36^ or may reappear in the SC after injury^37^. The presence of such guidance molecules or perhaps of the uninjured host RGC axons could account for the targeting of the transplant's axons in the brain if the ‘immature,' still-developing donor RGCs express molecules that allow them to respond to guidance cues along the host optic nerve. The exuberant arborization in these regenerating axons and potential mistargeting could be further studied by single-cell labelling or by use of RGCs extracted from Brainbow^38^ mice. How these donor cells would behave in the presence of an injured host optic nerve remains to be seen. Promising results with therapies after optic nerve crush showing improved visual function in test animals^39^,^40^,^41^ suggest that reinnervation from donor RGCs may likewise be possible in optic neuropathy models so long as donor RGC axons can navigate past the crush site, providing important areas for future investigation.

We have found that following intravitreal transplantation, donor RGCs respond to light stimulation of the acutely explanted host retina. The broad groups of light response characteristics include the ON, OFF and ON–OFF responses that in adult animals correlate with morphological differences in dendrite stratification^42^. The ON response of donor RGCs as well as steady firing at light offset in the OFF RGCs was noticeably more susceptible to adaptation than ON responses recorded from host RGCs at similar ages. In addition, compared with host RGCs, transplanted RGCs exhibit lower excitability upon current injection, indicative of immature electrical properties that could contribute to the weaker light responses. Mouse RGCs may not optimally form synapses with the rat host neurons, further reducing synaptic integration. Possibly the nascent synapses formed by donor RGCs in the host retina, recreating a new cross-species ON response circuit, are also weaker than their endogenous counterparts' established synapses, and the adaptation reflects declining synaptic strength or a changing balance of excitation and inhibition, as photoreceptor adaptation is not significant, as judged by the host RGC responses. Fine-tuned modulation of RGC physiology depends on precise centre–surround interactions^43^. The adaptation seen in the ON response could also be explained by a relatively stronger lateral inhibition from the new receptive field surround compared with the ON-excitation of the receptive field centre^44^. These interactions can be further investigated using more complex light stimulation paradigms to study centre–surround responses in the donor cells. In addition, an in-depth analysis of structure–function correlation in the transplanted RGCs will allow characterization of the types of synaptic connections made by the donor cells. A recent study has suggested that the number of ON–OFF RGCs decreases with age^45^, raising the possibility that the ON–OFF responses seen in our preliminary recordings could have been caught along the path to maturing into ON or OFF RGCs. With improved transplantation efficiency and longer times after transplantation, it will be useful to determine if the proportions of ON–OFF RGCs declines.

In conclusion, our findings offer a promising direction for retinal cell transplantation in diseases involving RGC degeneration. Our data demonstrate the potential for transplanted RGCs to repopulate and integrate into host retinas, and for such preparations to be used to determine whether transplanted cells retain their original physiological identities. Moreover, evidence of synaptic integration following cross-species transplantation of mouse RGCs into rat eyes strengthens the case for using allogeneic donor cells in cell replacement therapy models. Such transplants may be useful in disease models of glaucoma and optic nerve injury.

## Methods

### Animals

All use of animals conformed to the Association for Research in Vision and Ophthalmology Statement for the Use of Animals in Research and was approved by the Institutional Animal Care and Use Committee and the Institutional Biosafety Committee of the University of California, San Diego, and University of Miami. One- to three-month-old Sprague–Dawley rats, both male and female, were obtained from Harlan Laboratories. GFP mice were bred from the C57BL/6-Tg(CAG-EGFP)1Osb/J strain obtained from Jackson Laboratories. The animals used were randomly selected from groups of similarly aged animals. Because of the novelty of our experiments, no prior estimate of variance was available, so an a priori power analysis was not performed beforehand. However, adequate statistical analysis was performed for each experiment to permit assessment of statistical significance.

### RGC purification

RGCs were acutely purified from GFP mice between postnatal day 1 and 5 by sequential immunopanning with the CD90 (Thy1.2, AbD Serotec) antibody, yielding 99.5% pure RGCs^9^,^10^. Briefly, freshly extracted retinas were dissociated with papain (165 units; Worthington), followed by removal of macrophages and endothelial cells by immunopanning using anti-macrophage antibody (Accurate Chemical and Scientific Corporation; AIA31240). RGCs were selected from macrophage-depleted cell suspension using anti-CD90. Purified GFP-positive RGCs were re-suspended at a concentration of 10,000–20,000 cells per μl^−^ in serum-free, defined medium containing growth factors before *in vivo* transplantation within 1 h. In 70 experiments, remaining untransplanted donor cells were plated on PDL-/laminin-coated 24-well tissue culture plates in serum-free RGC growth medium^9^ and cultured for 3 days to evaluate survival of donor cells *in vitro* as an indicator of their viability. RGC growth medium was prepared with Neurobasal supplemented with insulin (5 μg ml^−1^; Sigma), sodium pyruvate (1 mM; Sigma), L-glutamine (1 mM; Sigma), triiodothyronine(T3; 40 ng ml^−1^; Sigma), *N*-acetyl cysteine (5 μg ml^−1^; Sigma), GS21 (1:100; GlobalStem), BDNF (brain-derived neurotrophic factor, 50 ng ml^−^; Peprotech), CNTF (ciliary neurotrophic factor, 10 ng ml^−^; Peprotech) and forskolin (5 mM; Sigma) as described^9^.

### *In vivo* RGC transplantation

GFP^+^ RGCs were transplanted intravitreally into randomly selected male and female rats as described^8^. Either 40,000 or 60,000 RGCs in 3–4 μl of serum-free medium were injected into the vitreous space of anaesthetized animals using a 31-G Hamilton syringe inserted into each eye. In some cases, including one set of experiments designed to evaluate donor axon growth into the optic nerve and brain, cells were injected into only one eye of the host animal. The animals were placed on a heating pad until awake.

### Electrophysiological recording of light responses

One to four weeks after transplantation, acute retinal explants were prepared as described previously^46^. Briefly, transplanted hosts were enucleated and the eyes were transferred into Ames' medium equilibrated with 5% CO_2_ at room temperature. An eye cup was dissected under sterile conditions and the retina removed from the eye cup, taking care to avoid damaging the tissues. The explanted retinas were prepared for electrophysiological recordings as described^47^. Briefly, acutely explanted retinas were treated with a mixture of collagenase (2,400 U ml^−1^) and hyaluronidase (600 U ml^−1^; both from Worthington Biochemical Corporation) in Ames' medium under 5% CO_2_, 95% O_2_ for 15 min in the dark. The explants were rinsed and then perfused in equilibrated Ames' before and after placing them in a recording chamber. Transplanted RGCs were identified by their GFP fluorescence and, for the remainder of the procedure, differential interference contrast microscopy was used with infrared illumination to avoid bleaching the photoreceptors. GFP^+^ RGCs were patched using pulled (Sutter P-97) glass micropipettes of 3–6 MΩ resistance, measured in the bath, under visualization with a camera (AxioCam MRm, Carl Zeiss) and monitor. The pipette solution was comprised of the following, in mM: 125 potassium gluconate, 2 CaCl_2_, 2 MgCl_2_, 10 EGTA, 10 HEPES, 2 sodium-ATP, 0.5 sodium-GTP and 8 biocytin; pH 7.2. After achieving whole-cell configuration, the donor cells had resting potentials ranging between −50 and −60 mV. The preparation was then further dark-adapted 5 min by switching off the lights and computer monitors. The retina was then stimulated with 1 s pulses of light (130 lux; Amprobe LM-200LED) from a white light LED driven by a stimulator (A-M Systems, Model 2100). Responses of patched cells to the light pulses were recorded in gap-free recording mode.

### Immunostaining

Retinas and, in some cases, optic nerves and brains from the host animals receiving transplants were dissected, fixed for 30 min in 4% paraformaldehyde, and either mounted directly or transferred through a sucrose gradient for cryoprotection, and placed into Optimal Cutting Temperature compound for cryopreservation. Eye cups, optic nerves and brains cryopreserved in Optimal Cutting Temperature compound were sectioned at 30 μm (retinas and optic nerves) or 50 μm (brains) with a cryostat. Care was taken to ensure that the brain tissues from all samples were processed and sectioned similarly. Retinas used for recordings were fixed for 30 min followed by treatment with Alexa 546-conjugated streptavidin (1:1,000; ThermoFisher Science, S-11225) to label the biocytin in patched cells. Retinas and sections were blocked with 5% serum and permeabilized with 0.2% Triton X-100 before staining with anti-GFP (1:1,000; Millipore, MAB10145) anti-Brn3a (1:100; Millipore MAB1585), anti-synaptophysin (1:500; SySy, 101004) or PSD95 (1:100; Pierce, MA1–045) antibody overnight at 4 °C. Fluorophore-conjugated goat secondary antibodies were used to then label the primary antibodies. Stained retinal explants, optic nerve- and brain sections were imaged using confocal microscopy (Leica TCS SP5). For synaptic puncta measurements, confocal images were analysed using Volocity software (Perkin Elmer) to look for coincidence between each of the labelled populations, GFP, PSD95 and synaptophysin.

### Cell counting and data analysis

In 12 of 152 tested retinas, the numbers of retained GFP^+^ RGCs in the recipient whole-mounted retinas were counted using the automated counting function on FIJI software^48^. Briefly, the images were thresholded to obtain the most representative image and particles were analysed with circularity range of 0.5–1.0 as a cutoff. A subset of the automated counts was validated by manual counting to minimize error. In samples with good DAPI staining (*n*=6), cells with healthy DAPI-stained nuclear morphology were manually counted on FIJI, moving plane by plane through confocal stacks to avoid misinterpretation; healthy cells were defined as large and round, regular and neither fragmented nor pyknotic. Similarly, cells with visible axon-like processes were separately counted. Retinas were divided into equal radial quadrants, with the ONH at the centre, to compare GFP^+^ cell density with neurite growth within each quadrant. Neurites longer than a single-cell body length were counted and, for axon orientation, only defined, long axons were counted. The data were correlated using Excel (Microsoft) and Sigmaplot (Systat Software Inc.) to obtain *r*-values. Numbers of cells with and without processes were compared using a two-sided *t*-test assuming unequal variance. Three successfully transplanted eyes were fixed and processed for sectioning. Parallel cultures were made by plating left-over donor cells through the same Hamilton syringe used for transplantation, mimicking the transplant condition. The cultures were viewed after 3 days *in vitro* and both cell morphology and the presence of neurites were noted. Cultures with fewer than ∼75% of RGCs exhibiting healthy morphology were labelled poor survival cultures. Although the investigators were not blinded during experiments, these survival observations were recorded before explanting transplanted retinas to preclude biased assessment of transplant success. To study dendrite architecture, 94 individual GFP^+^ cells in the transplant recipient retinas were imaged using confocal microscopy at high laser power and their neurites traced using Photoshop (Adobe). Dendritic field diameters were measured as the diameters of circles with the same area as polygons connecting the farthest dendrite ends of each cell. The Sholl analysis plug-in for FIJI was used to analyse intercepts of individual donor RGCs with the concentric circles and determine the circle diameter that had the maximal (peak) number of intercepts. To estimate the number of axons extending into the host optic nerve, we counted the number of GFP^+^ axons within a defined length of the stained optic nerve sections and extrapolated the total number in the corresponding cylindrical segment of the optic nerve. This analysis was done on two separate sections from each of the three host optic nerves analysed.

## Additional information

**How to cite this article:** Venugopalan, P. *et al.* Transplanted neurons integrate into adult retinas and respond to light. *Nat. Commun.* 7:10472 doi: 10.1038/ncomms10472 (2016).

## Supplementary Material

Supplementary Information Supplementary Figures 1-4

## Figures and Tables

**Table 1:** Healthy donor cell population may contribute to successful transplantation.

	**Total transplantations (# of retinas)**	**Successful transplants (# (%))**
Good survival in parallel cultures	14	6 (42.8)*
Poor survival in parallel cultures	56	0 (0)*
No parallel cultures made	82	9 (10.9)

^*^P<0.0001 by Fisher's Exact test, two-tailed.

When success of transplantations was compared with quality of donor cells in parallel cultures, success was higher when parallel cultures of donor cells were healthy.

**Table 2:** The total number of synaptic puncta associated with GFP^+^ RGCs in three separate sections.

**Image**	**Total synaptic puncta**	**# Associated with GFP**	**% Of total associated with GFP**	**Total cells in section**	**# Of synapses per cell in section**
1	1,358	13	0.96	5	2.6
2	1,279	39	3	11	3.54
3	1,595	51	3.12	13	3.92
Average	1,410	34	2.36	∼9	3.35

GFP, green fluorescent protein; RGC, retinal ganglion cell.
