# Supplementary material for: Transplanted neurons integrate into adult retinas and respond to light
Source: Nat Commun. 2016 Feb 4;7:10472. doi: 10.1038/ncomms10472 (PMC4742891; doi:10.1038/ncomms10472)
Supplement: Supplementary Information — Supplementary Figures 1-4 [file ncomms10472-s1.pdf]

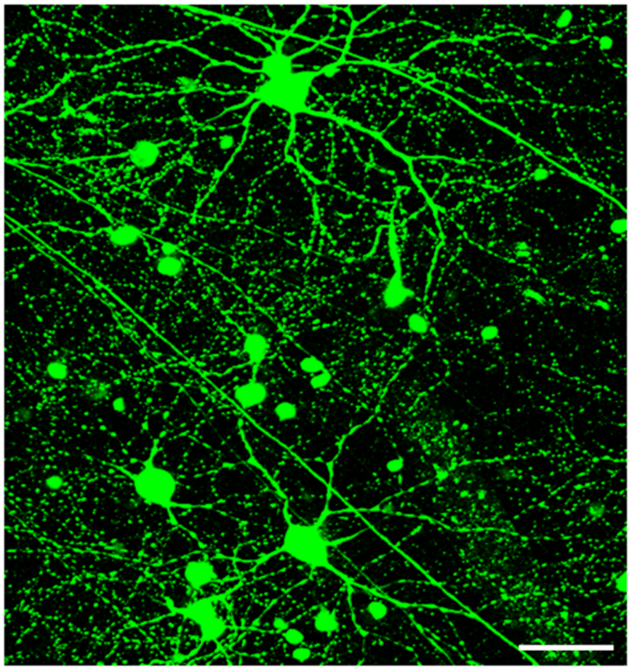

Supplementary Figure 1: GFP$^+$ transplanted RGCs grow extensive dendrite-like neurites into the inner plexiform layer. Image of whole-mounted transplant recipient retina taken at high laser power showing bright GFP$^+$ dendrites extending from many of the visible GFP$^+$ RGCs. Scale bar 50 µm.

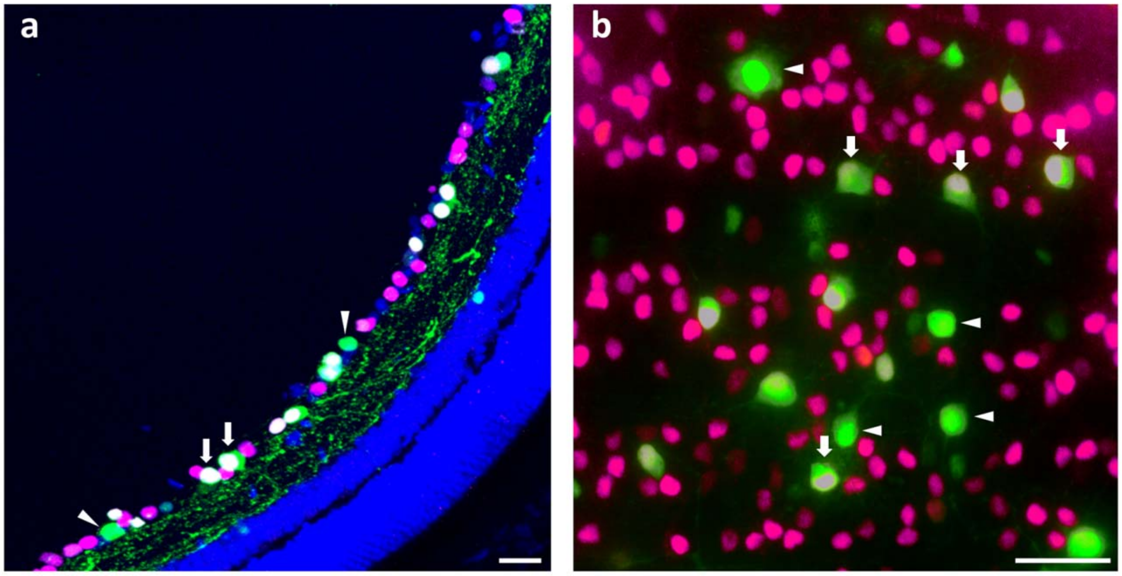

Supplementary Figure 2: Transplanted GFP+ RGCs express Brn3a similar to known Brn3a expression patterns. Representative images from transplant recipient retinal section (a) and whole mount (b) stained for GFP (green), Brn3a (magenta) and DAPI (blue). Arrowheads highlight GFP$^+$ RGCs that are Brn3a, while the arrows highlight some GFP$^+$ RGCs that are Brn3a$^+$. Scale bar 50 µm.

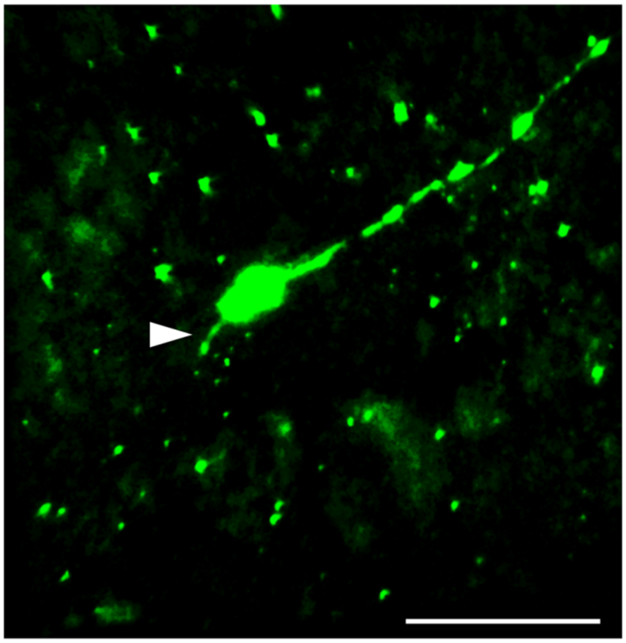

Supplementary Figure 3: Transplanted RGCs extend growth cones growing towards the optic nerve head. High magnification image (100X) of growth cone at the end of a GFP^+ axon one week post-transplantation. Arrowhead highlights the presence of a filopodium-like structure extending from the leading edge of the growth cone. Scale bar 10 µm.

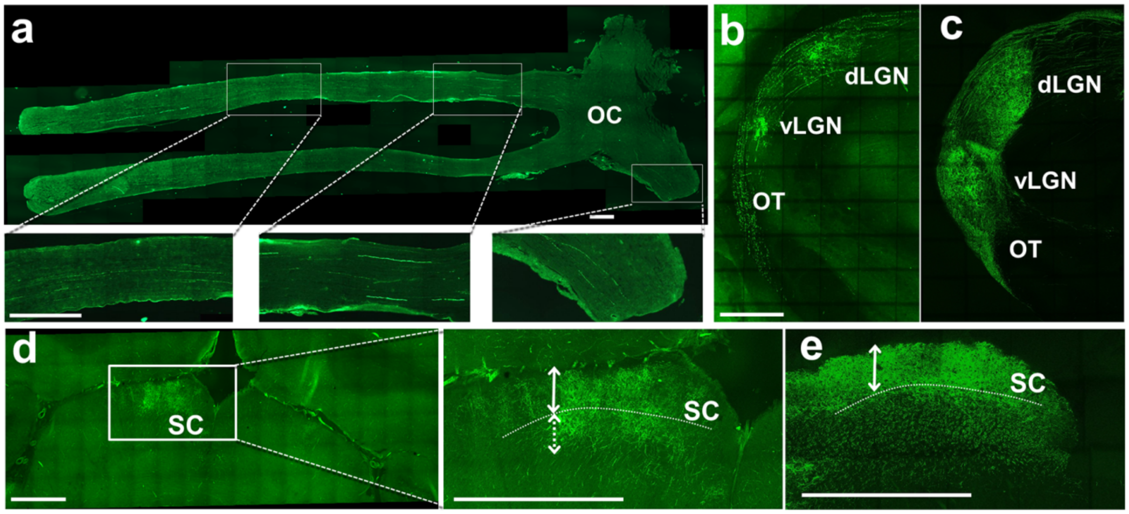

Supplementary Figure 4: Transplanted RGCs extend axons through host optic nerves and into known visual targets in the brain. Optic nerves and brains of host animals, fixed and sectioned one month after unilateral transplantation, contain labeled fibers.. (**a**) In fixed optic nerve sections, GFP^+ axons extend to the optic chiasm (OC) and cross over. (**b**) These axons are visible within the optic tract (OT) and in known RGC targets in the brain, such as nuclei in the ventral and dorsal lateral geniculate nucleus (LGN) (**c**) For comparison, RGC axon tracts in age matched control brains labeled by intravitreal injection of AAV-GFP (**d**) Axons terminals exhibiting an immature innervation pattern in deeper layers of the superior colliculus (SC) of host animals. (**e**) AAV-GFP labeled RGC axon innervation of superficial layers of the superior colliculus in age-matched control animals is more restricted to those layers. Medial-lateral axis of the SC from right to left. Scale bars: 500 µm
